# Novel TNF receptor-1 inhibitors identified as potential therapeutic candidates for traumatic brain injury

**DOI:** 10.1186/s12974-018-1200-y

**Published:** 2018-05-22

**Authors:** Rachel K. Rowe, Jordan L. Harrison, Hongtao Zhang, Adam D. Bachstetter, David P. Hesson, Bruce F. O’Hara, Mark I. Greene, Jonathan Lifshitz

**Affiliations:** 1 BARROW Neurological Institute at Phoenix Children’s Hospital, Phoenix, AZ USA; 2 Department of Child Health, University of Arizona College of Medicine—Phoenix, Phoenix, AZ USA; 3 Phoenix Veteran Affairs Healthcare System, Phoenix, AZ USA; 4 Basic Medical Sciences, University of Arizona College of Medicine—Phoenix, Phoenix, AZ USA; 5 University of Pennsylvania Perelman School of Medicine, Philadelphia, PA USA; 6 Sanders-Brown Center on Aging, Spinal Cord and Brain Injury Research Center, and Department of Neuroscience, University of Kentucky, Lexington, KY USA; 7 Department of Biology, University of Kentucky, Lexington, KY USA

**Keywords:** Diffuse brain injury, Midline fluid percussion, Mouse, Concussion, Cytokines, Tumor necrosis factor

## Abstract

**Background:**

Traumatic brain injury (TBI) begins with the application of mechanical force to the head or brain, which initiates systemic and cellular processes that are hallmarks of the disease. The pathological cascade of secondary injury processes, including inflammation, can exacerbate brain injury-induced morbidities and thus represents a plausible target for pharmaceutical therapies. We have pioneered research on post-traumatic sleep, identifying that injury-induced sleep lasting for 6 h in brain-injured mice coincides with increased cortical levels of inflammatory cytokines, including tumor necrosis factor (TNF). Here, we apply post-traumatic sleep as a physiological bio-indicator of inflammation. We hypothesized the efficacy of novel TNF receptor (TNF-R) inhibitors could be screened using post-traumatic sleep and that these novel compounds would improve functional recovery following diffuse TBI in the mouse.

**Methods:**

Three inhibitors of TNF-R activation were synthesized based on the structure of previously reported TNF CIAM inhibitor F002, which lodges into a defined TNFR1 cavity at the TNF-binding interface, and screened for in vitro efficacy of TNF pathway inhibition (IκB phosphorylation). Compounds were screened for in vivo efficacy in modulating post-traumatic sleep. Compounds were then tested for efficacy in improving functional recovery and verification of cellular mechanism.

**Results:**

Brain-injured mice treated with Compound 7 (C7) or SGT11 slept significantly less than those treated with vehicle, suggesting a therapeutic potential to target neuroinflammation. SGT11 restored cognitive, sensorimotor, and neurological function. C7 and SGT11 significantly decreased cortical inflammatory cytokines 3 h post-TBI.

**Conclusions:**

Using sleep as a bio-indicator of TNF-R-dependent neuroinflammation, we identified C7 and SGT11 as potential therapeutic candidates for TBI.

**Electronic supplementary material:**

The online version of this article (10.1186/s12974-018-1200-y) contains supplementary material, which is available to authorized users.

## Background

There are approximately 1.7 million TBIs in the USA annually, with approximately 1.36 million that seek medical treatments [1]. The primary injury caused by a TBI occurs at the time of the mechanical impact, with limited possibilities to mitigate this damage; however, secondary injury processes, such as inflammation, oxidative stress, and excitotoxicity, amplify the primary damage and represent plausible targets for pharmaceutical intervention [2]. The development of an effective pharmacological therapy for acute TBI could prevent, restrict, or reverse the emergence of chronic post-traumatic morbidities. Such therapy may also require a systematic pre-clinical paradigm to translate pharmacological neuroprotection into improving neurological performance, as proposed by Janowitz and Menon [3].

Following TBI, the pathophysiological inflammation as part of secondary injury processes can contribute to worsening outcome [4, 5], particularly with unregulated inflammatory cytokine signaling [4–6]. Tumor necrosis factor (TNF)-α has central nervous system function by contributing to the activation of microglia and astrocytes and release of inflammation mediating cytokines and can influence blood-brain barrier permeability and synaptic plasticity [7]. TNF functions through interaction with two members of the TNF-receptor (TNFR) family, TNFR1 and TNFR2. TNFR1 has been associated with inflammation and degeneration, whereas TNFR2 has been identified as neuroprotective [8, 9], for review see [10]. For this reason, we chose to spare the effects of TNF through TNFR2 signaling and selectively inhibit TNF function through TNFR1.

In vitro studies have revealed complex and divergent TNFR signaling pathways that account for TNF’s ability to induce cell death, co-stimulation, and cell activation [11]. TNFR signaling results in the phosphorylation and degradation of the inhibitor of NF-ĸB (IĸB), which allows for the translocation of NF-ĸB to the nucleus where it binds DNA and functions as a transcription factor [11]. Thus, TNF-induced NF-ĸB is a master regulator of inflammation since NFĸB is a global activator of pro-inflammatory cytokines, chemokines, and their receptors [11].

TNF-α is pleiotropic in its actions regarding the regulation of inflammation. Relevant to the current study, substantial evidence also supports the involvement of TNF-α in the physiological process of sleep [12–16]. An increase in sleep is temporally associated with the acute increase in inflammation following TBI [17]. Therefore, sleep can be used as a sensitive bio-indicator of the ability of novel TNFR1 inhibitor compounds to modulate TBI-induced neuroinflammation and behavioral phenotypes. Herein, we report that the novel allosteric modulators of TNFR1 inhibitors improved functional recovery following diffuse TBI in the mouse. In addition, we validated sleep as a bio-indicator of TNFR1-dependent neuroinflammation, which could be applied as a minimally invasive approach to show therapeutic efficacy in future clinical studies.

## Methods

### Study design

In a controlled laboratory experiment, this study tested a refined pre-clinical strategy to identify and confirm the utility of experimental compounds with therapeutic potential for treating acute TBI pathophysiology. All animal studies were conducted in accordance with the guidelines established by the internal IACUC (Institutional Animal Care and Use Committee) and the NIH guidelines for the care and use of laboratory animals. Studies are reported following the ARRIVE (Animal Research: Reporting In Vivo experiments) guidelines [18]. Randomization of animals was achieved by assigning animals to treatment groups before the initiation of the study to ensure equal distribution across groups. A power analysis was performed to calculate group sizes that enable statistically robust detection of injury-induced deficits while minimizing the number of animals needed. This calculation was based on preliminary data and previously published work from our group. Data collection stopped at pre-determined final endpoints based on days post-injury for each animal. Animals were excluded from the study if post-operative weight decreased by 15% of pre-surgical weight (*n* = 3), or pre-injury baseline rotarod score was not met (*n* = 5). All animal behavior was scored by investigators blinded to the treatment groups and blinded to drug groups. Data sets were screened using the extreme studentized deviate method for significant outliers. One significant outlier treated with C7 was found and excluded from the cytokine analyses.

### Compound design and synthesis

F002 was the first highly active lead in a series of rigid propeller-like compounds identified from commercial databases by docking to a cavity-induced allosteric modifier (CIAM) algorithm identified pocket on TNFR1. Further modeling efforts combined the propeller-like arrangement of the aryl groups with the goal of improved solubility, using a propane diol backbone. These changes led to two novel compounds, C7 and SGT11. C7 had a slightly better SYBYL-X docking score (2.68), but possessed a higher molecular weight (567.99) and more lipophilicity, with a CLogP score of 5.45. Removal of the benzyl alcohol acetates from C7 yielded the corresponding bis-benzyl alcohol SGT11 with significantly improved SYBYL-X score (6.15), reduced molecular weight, and a 2-log drop in CLogP. The total calculated surface area for all of the molecules were similar and ranged from 75.53 to 87.67, with SGT11 being smallest. The compounds were synthesized by Shanghai Medicilion (Shanghai, China).

### In vitro screening

#### NFκB activity reporter gene assay

To study TNFα-mediated NFκB activity, a luciferase reporter system was established. The stable cell line A549-LUC-14 was derived from human A549 cells with chromosomal integration of a luciferase reporter construct regulated by six copies of the NFκB response element. This clonal cell line was obtained by co-transfection of pNFκB-TA-luc and pFLAG-TNFR1-neo followed by G418 selection at 800 μg/ml. A549-LUC-14 cells were seeded, at 3000 cells per well, in a 96-well flat-bottom cell culture plate. After incubation at 37 °C for 24 h, cells were changed into fresh medium with indicated compounds for 1 h pre-incubation. Then, 50 μl of medium containing TNF-α or control was added to each well for a 4-h incubation at 37 °C. The final TNF-α was 5 ng/ml. After 4 h, media were removed and cells were gently washed twice with 200 μl DPBS. Twenty-five microliters of 1× reporter lysis buffer was added to each well at room temperature and incubated for 5 min, followed with incubation at − 80 °C for 1 h. The plate was thawed at room temperature, and 20 μl of each sample was transferred into an opaque 96-well plate to measure luminescence using a Luminoskan Ascent luminometer (Thermo Labsystems, Franklin, MA).

#### Western blot

L929 cells were grown on tissue culture plastic in complete DMEM supplemented with 5% FBS, non-essential amino acids, and glutamine. To obtain cell lysates for western blot, cells treated with TNF-α and inhibitors were lysed in a RIPA-based buffer consisting of 20 mM Tris-HCl (pH 7.4), 150 mM NaCl, 1 mM EDTA, 1 mM EGTA, 1% Nonidet P-40, 1% sodium deoxycholate, 10 mM NaF, 1 mM sodium orthovanadate, and Complete Mini Protease Inhibitors (Roche Diagnostics) on ice for 15 min and lysates were clarified by centrifugation (16,000 × *g*) for 10 min. Supernatant was used for western blot analysis after separation by SDS-PAGE and was subsequently transferred to a nitrocellulose membrane. Membranes were subsequently blocked with 5% non-fat dry milk in PBS buffer for 1 h at room temperature. Membranes were then washed and incubated with primary antibodies (Cell Signaling Technology, 1:10,000 dilution), followed by the appropriate HRP-conjugated secondary antibody. The membrane was then treated with chemiluminescent HRP substrate (Millipore) and exposed to Hyblot CL autoradiography film (Denville Scientific Inc.).

### Animals

Male C57BL/6 mice (20–24 g) (Harlan Laboratories, Inc., Indianapolis, IN) were used for all experiments (*n* = 103). Mice were housed in a 14-h light/10-h dark cycle at a constant temperature (23 °C ± 2 °C) with food and water available ad libitum according to the Association for Assessment and Accreditation of Laboratory Animal Care International. All mice used in this study were singly housed. Mice were acclimated to their environment following shipment for at least 3 days prior to any experiments. After surgery, mice were evaluated daily during post-operative care via a physical examination and documentation of each animal’s condition. Animal care was approved by the Institutional Animal Care and Use Committees at the University of Arizona (Tucson, AZ).

### Midline fluid percussion injury (mFPI)

Since human TBI is a markedly heterogeneous disease, no single animal model of TBI can reproduce the entire spectrum of clinical TBI features and symptoms. For this study, we used the midline fluid percussion injury (mFPI) model for its clinical relevance (for review see Lifshitz et al.) [19]. mFPI can model diffuse TBI resulting in acute behavioral deficits and late-onset behavioral morbidities in the absence of a focal component, gross histopathology, and cavitation, as seen with controlled cortical impact (CCI) [19, 20]. In the current study, adult male mice (2 months of age) were subjected to midline fluid percussion injury consistent with methods previously described [17, 21–25]. Group sizes are indicated in the “Results” section and figure legends for individual studies. Mice were anesthetized using 5% isoflurane in 100% oxygen for 5 min, and the head of the mouse was placed in a stereotaxic frame with continuously delivered isoflurane at 2.5% via nosecone. While anesthetized, body temperature was maintained using a Deltaphase® isothermal heating pad (Braintree Scientific Inc., Braintree, MA). A midline incision was made exposing bregma and lambda, and fascia was removed from the surface of the skull. A trephine (3 mm outer diameter) was used for the craniotomy, centered on the sagittal suture between bregma and lambda without disruption of the dura. An injury cap prepared from the female portion of a Luer-Loc needle hub was fixed over the craniotomy using cyanoacrylate gel and methyl-methacrylate (Hygenic Corp., Akron, OH). The incision was sutured at the anterior and posterior edges, and topical Lidocaine ointment was applied. The injury hub was closed using a Luer-Loc cap, and mice were placed in a heated recovery cage and monitored until ambulatory before being returned to their piezoelectric sleep cage.

For injury induction 24 h post-surgery (approximately 11:00), mice were re-anesthetized with 5% isoflurane delivered for 5 min. The cap was removed from the injury hub assembly, and the dura was visually inspected through the hub to make sure it was intact with no debris. The hub was then filled with normal saline and attached to an extension tube connected to the male end of the fluid percussion device (Custom Design and Fabrication, Virginia Commonwealth University, Richmond, VA). An injury of moderate severity for our injury model (1.1–1.2 atm) was administered by releasing the pendulum onto the fluid-filled cylinder. Sham-injured mice underwent the same procedure except the pendulum was not released. Mice were monitored for the presence of a forearm fencing response, and righting reflex times were recorded for the injured mice as indicators of injury severity [26]. The righting reflex time is the total time from the initial impact until the mouse spontaneously rights itself from a supine position. The fencing response is a tonic posturing characterized by extension and flexion of opposite arms that has been validated as an overt indicator of injury severity [26]. The injury hub was removed, and the brain was inspected for uniform herniation and integrity of the dura. The dura was intact in all mice, so none were excluded as technical failures. The incision was cleaned using saline and closed using sutures. Diffuse brain-injured mice had righting reflex recovery times greater than 5 min, less than 11 min, and a positive fencing response. Sham-injured mice recovered a righting reflex within 20 s. After spontaneously righting, mice were placed in a heated recovery cage and monitored until ambulatory (approximately 5 to 15 additional min) before being returned to their individual sleep cage (vide infra). Adequate measures were taken to minimize pain or discomfort [27].

### Pharmacological treatment

Mice were treated with vehicle (10% DMSO), C7, SGT-11, or F002. Compounds were administered intraperitoneally at (20 mg/kg) or (2 mg/kg) immediately following injury. In the subsequent functional and histological studies, compounds (2 mg/kg) were administered intraperitoneally twice: immediately following the injury and 24 h post-injury. Dosing was selected with regard to published studies using F002 in mice [28] and the solubility of the novel compounds. High doses (20 mg/kg) were maximum solubility, whereas low doses were one tenth of that dose (2 mg/kg).

### Sleep recordings

The non-invasive sleep cage system (Signal Solutions, Lexington, KY) used in this study consisted of 16 separate units that simultaneously monitored sleep and wake states, as previously published [22, 29, 30]. Each cage unit housed a single mouse inside 18 × 18-cm walled compartments with attached food and water structures [29]. The cages had open bottoms resting on polyvinylidine difluoride (PVDF) sensors serving as the cage floor [29]. The non-invasive high-throughput PVDF sensors were coupled to an input differential amplifier, and pressure signals were generated and classified by the classifier as motions consistent with either activity related to wake or inactivity and regular breathing movements associated with sleep [22, 29]. Briefly, sleep was characterized primarily by periodic (3 Hz) and regular amplitude signals recorded from the PVDF sensors, which is typical of respiration from a sleeping mouse. In contrast, signals characteristic of wake were both the absence of characteristic sleep signals and higher amplitude, irregular signals associated with volitional movements, even during quiet wake. The piezoelectric signals in 2-s epochs were classified by a linear discriminant classifier algorithm based on multiple signal variables to assign a binary label of “sleep” or “wake” [29]. Mice sleep in a polycyclic manner (often more than 40 sleep episodes per hour if short arousals are recorded) [31], and therefore, mouse sleep was quantified as the minutes spent sleeping per hour, presented as a percentage for each hour. Data collected from the cage system were binned over specified time periods (e.g., 1 h) using the average of percent sleep, as well as binned by length of individual bouts of sleep. Where applicable, sleep metrics were compared between 6 h of light and 6 h of dark.

### Rotarod

Sensorimotor function was assessed using the Economex Rotarod system from Columbus Instruments (Columbus, OH). Mice were acclimated 3 days prior to surgery/injury. The mice were placed on the stationary rod and allowed to explore for 30 s. Following exploration, the mice were placed on the rod at a constant speed of 5 revolutions per minute (rpm). If the mouse fell off the rod, it was placed back on the rod and the timer was restarted (until the mice could walk 15 s at 5 rpm). Next, mice were placed on the rod with a rotation of 5 rpm and an acceleration of 0.2 rpm/s. The trial ended when the mouse fell off the rod; after two trials, the acclimation period ended. The testing phase occurred over 3 consecutive days prior to surgery/injury (last test before surgery was recorded as baseline) and 1, 3, 5, and 7 days post-injury (DPI). For the post-injury testing phase, mice were placed on the stationary rod and the instrument was started at 5 rpm with an acceleration of 0.2 rpm/s. Two trials were run back to back, after which mice were returned to holding cages. After 10 min, mice performed a third trial. The times from the best two trials were averaged to generate a latency to fall from the rotating rod for each mouse.

### Neurological severity score (NSS)

Post-traumatic neurological impairments were assessed at 24 h post-injury using an eight-point NSS paradigm implemented from those previously used in experimental models of TBI [32–35]. One point was given for failure on an individual task, and no points were given if a mouse completed a task successfully. Mice were observed for hindlimb flexion, startle reflex, and seeking behavior (presence of these behaviors was considered successful task completion). Mice traversed in sequence, 3-, 2-, and 1-cm beams. The beams were elevated, and mice were given 1 min to travel 30 cm on the beams. The task was scored as a success if the mouse traveled 30 cm with normal forelimb and hindlimb position (forelimb/hindlimb did not hang from the beam). Mice were also required to balance on a 0.5-cm beam and a 0.5-cm round rod for 3 s in a stationary position with front paws between hind paws. Non-parametric data are presented as a composite score ranging from zero to eight representing performance on all tasks combined. High final NSS scores were indicative of task failures and interpreted as neurological impairment.

### Novel object recognition (NOR)

Cognitive function was tested using the NOR test as previously published [22, 36, 37]. The test consisted of three phases: habituation, training, and testing. On day 6 post-injury, mice were placed in an open field (21 × 21 × 42 cm) for 1 h of habituation. Mice were removed and two identical objects were placed in opposing quadrants of the field for the training phase. Mice were placed in the center of the open field and given 5 min to explore the objects. Following training, mice were returned to their individual sleep cages. Testing began 4 h after training. One familiar object was placed in an original location, and one novel object was placed in the opposing quadrant of the open field. Mice were placed into the center and given 5 min to explore. For testing, the times spent actively investigating the novel and familiar object were quantified. Investigation of an object included sniffing, touching, or climbing onto an object while the mouse was facing the object. If an animal climbed onto an object and sniffed into the air, this time was not calculated into the exploration of the novel object. Testing data are reported as the percentage of total investigation time spent with each object and as a discrimination index (DI) in which DI = (*T*_novel_−*T*_familiar_)/(*T*_novel_ + *T*_familiar_). A discrimination index of zero indicates no object preference, and positive values show preference for the novel object.

### Tissue preparation for cytokine measurement and immunofluorescence

At selected time points (3 h or 7 days) post-injury or sham operation, mice were given an overdose of sodium pentobarbital (i.p.) and transcardially perfused with 4% paraformaldehyde after a phosphate-buffered saline (PBS) flush. For cytokine measurement, brains were dissected on ice and snap frozen in liquid nitrogen and then stored at − 80 °C until used. For immunofluorescence, brains were removed and placed in 4% paraformaldehyde overnight. Brains were immersed in serial dilutions (15 and 30%) of sucrose for 24 h each. The brains were removed from the 30% sucrose and frozen at − 20 °C. After freezing, brains were cryosectioned in the coronal plane at 20 μm, mounted onto glass slides, and stored at − 80 °C.

### Cytokine measurement

The protein levels of a panel of inflammatory cytokines were measured in the neocortex by Meso Scale Discovery (MSD) multiplex immunoassay (sector imager 2400, Meso Scale Discovery; Gaithersburg, MD) as previously described [38]. Brain cortex was homogenized using high shear homogenizer (Omni TH115), in a 1:10 (*w*/*v*) of ice-cold lysis buffer consisting of PBS containing 1 μg/ml Leupeptin, 1 mM PMSF, and 1 mM EDTA. The cortical homogenate was centrifuged at 14,000 × *g* for 20 min at 4 °C in a microcentrifuge. Fifty microliters of the resulting supernatant was loaded per well of the custom MSD plate, and cortical cytokine levels were determined by MSD assay (Mouse Proinflammatory 7-Plex Ultra-Sensitive (K15012C)). Cytokine levels in the cortex were normalized to the total amount of protein in the sample loaded as determined by BCA Protein Assay (Pierce).

### Iba-1 immunofluorescence

Slides were removed from − 80 °C, placed in an oven at 60 °C for approximately 4 h, and then rinsed three times for 5 min each in PBS. Next, the slides were incubated in 4% goat serum blocking solution for 1 h. Slides were incubated with the primary antibody (rabbit anti-ionized calcium-binding adaptor molecule 1, IBA-1; 1:1000, Item # 0199-19741, Wako Chemicals, Richmond, VA) and stored at 4 °C overnight. Slides were rinsed three times in PBS and the secondary antibody (biotinylated horse anti-rabbit; 1:250, Vector Laboratories, Burlingame, CA) was added and slides were incubated on a rocker at room temperature for 1 h. Slides were washed in PBS three times for 5 min each, tertiary stain was applied (streptavidin Alexa© Fluor 594; 1:1000, Jackson Immunoresearch, Westgrove, PA), and slides were incubated for 1 h at room temperature. Lastly, slides were rinsed three times in PBS and coverslipped with anti-fade medium (Fluoromount G; Southern Biotech, Birmingham, AL).

### Microglial identification and quantification

Stained sections (four sections per animal; *n* = 8 per group) were analyzed following Iba-1 staining to determine the proportion of microglial morphologies post-injury. The area of interest, the primary somatosensory barrel fields (S1BF), was chosen based on previous work demonstrating a focus of neuropathology and microglial activation in the S1BF following midline fluid percussion brain injury in the rodent [39–42]. Sections were screened using a Zeiss (AXIO imager A2) microscope with an attached digital camera (AxioCam MRc5). Images were captured with proprietary Zen software (Carl Zeiss, Germany) at × 20 magnification. The area of interest was examined in both hemispheres and in two different coronal sections: an anterior section (~Bregma − 1.555 mm) and a posterior section (~Bregma − 2.255 mm). A total of four photos per brain (*n* = 8 animals per group) per area of interest were analyzed. Photomicrographs were analyzed using Image J software (National Institutes of Health, Bethesda, MD). On each photomicrograph, 250,000 pixel^2^ grid lines were placed and quantification was limited to four pre-defined boxes. Microglia within these boxes were classified by a blinded investigator as either having ramified (small soma, high defined processes) or activated (hypertrophied soma, fewer processes) morphologies. A minimum of 50 microglia were counted per section per region. Sham treatment groups were combined.

### Statistical analysis

Results are shown as mean ± SEM and analyzed using GraphPad Prism 6, with statistical significance assigned at the 95% confidence level (*α* < 0.05), unless otherwise indicated. For each test, sham animals receiving each treatment were compared. No differences were detected between sham groups (treated and vehicle), so shams were combined from each treatment. Percent sleep and differences in rotarod performance were analyzed using a repeated measure two-way analysis of variance (ANOVA) followed by Tukey’s multiple comparisons test. Improvement on the rotarod was analyzed using a one-way ANOVA with a Sidak’s multiple comparisons test. Cumulative sleep measured in minutes and cytokine levels were analyzed with a one-way ANOVA followed by Tukey’s multiple comparisons test. Non-parametric Kruskal-Wallis tests implemented in the statistical program R [43] were used to investigate if median NSS scores differed among groups (including shams) across and within injury-specific time points. If Kruskal-Wallis tests were significant (*p* < 0.05), a post hoc Dunn test implemented in the R package DescTools [44] was performed with *p* values adjusted using a Bonferroni correction for multiple comparisons to determine which groups differed. Identical analytical methods were also used to investigate if median NSS scores differed among injury-specific time points without considering a group effect. Proportional differences in microglia were compared using a one-way ANOVA. For all parametric analyses, the assumption that data were normally distributed was verified using density and q-q plots and Shapiro-Wilk’s tests to ensure the validity of the analytical approaches used. Resulting test values are included in the figure legends.

## Results

### Novel TNF-R1 inhibitors demonstrate target engagement by blocking TNF-R1 signaling pathways

**Fig. 1:**
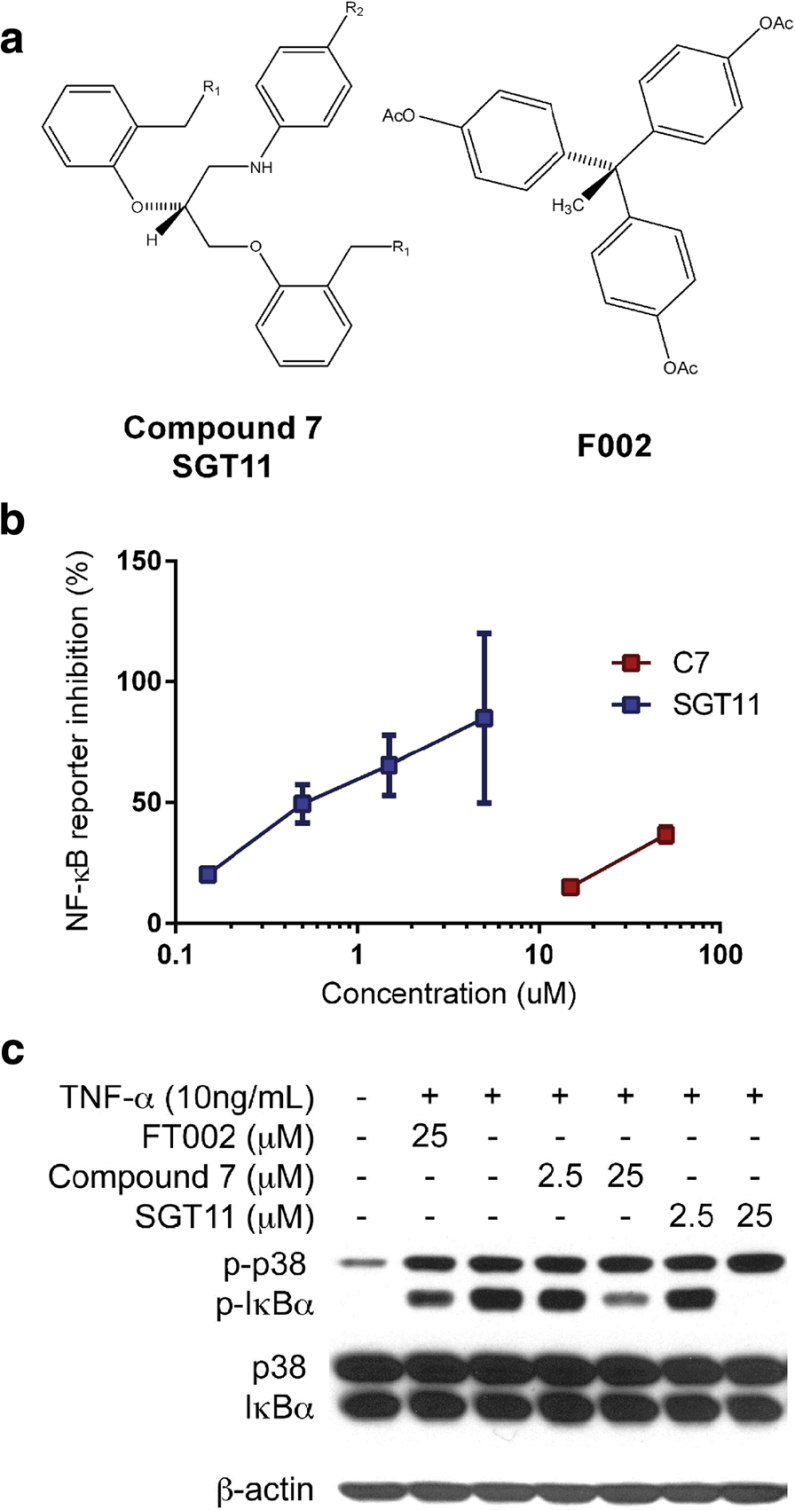
Novel TNF-R1 inhibitors demonstrate target engagement by blocking TNF-R1 signaling pathways. **a** Molecular structures of experimental compounds (C7 and SGT11) differ at R_1_ (C7 = OAc, SGT11 = OH), R_2_ = CF_3_. **b** C7 and SGT11 were tested to inhibit the TNFα-induced luciferase reporter gene expression controlled by NF-κB promoter. The percentage of inhibition of TNFα-induced reporter gene expression is shown. **c** Inhibition of TNF-α-induced phosphorylation of IκB in murine fibroblast L929 cells. The cells were pretreated with inhibitors for 1 h and stimulated by human TNFα at 5 ng/ml for 5 min. Phosphorylation and total protein levels of IκB and p38 were examined by western blot. The data represent a typical result derived from several experiments

We have previously reported a novel TNFR1 inhibitor, F002, which is a cavity-induced allosteric modifier (CIAM) of TNFR1 that inhibits TNF-α binding to TNFR1 and subsequent pathway activation [45]. To extend properties of F002, two new analogues were rationally designed and synthesized by Shanghai Medicilon (Shanghai, China). The two new compounds, called C7 and SGT11, differed in the R1 position (SGT11, R1 = OH; C7, R1 = OAc; Fig. 1a) and efficiently inhibited TNF pathway activation (Fig. 1b, c). CIAM compounds concentration-dependently inhibited NF-κB activation as demonstrated by the western blots of IκBα phosphorylation.

Upon binding to TNFRs, TNFα induces inflammation through the activation of NFκB and p38 MAPK signaling pathways. The novel CIAM compounds were tested for activity to inhibit TNFα-induced NF-κB signaling and p38 MAPK signaling. An NF-κB reporter gene system was generated in the human A549 cell line by chromosomal integration of a luciferase reporter construct under the transcriptional control of the NF-κB response element. In this system, TNFα treatment activates NF-κB and results in luciferase expression. As shown in Fig. 1b, both C7 and SGT11 inhibited the luciferase activity concentration-dependently. However, SGT11, which had an IC50 of 5.5 μM, showed greater inhibition than C7, which did not reach 50% inhibition at 50 μM. These compounds, which were confirmed to inhibit NF-κB phosphorylation (Fig. 1c), were selected to move to in vivo testing. At 25 μM, F002 minimally inhibited the TNF-α-induced phosphorylation of IκBα (p-IκBα). In contrast, at 25 μM, both C7 and SGT11 limited p-IκBα phosphorylation (Fig. 1c). None of these inhibitors displayed significant activity on TNF-α-induced p38 phosphorylation.

### Novel TNF-R1 inhibitors modulated post-traumatic sleep

**Fig. 2:**
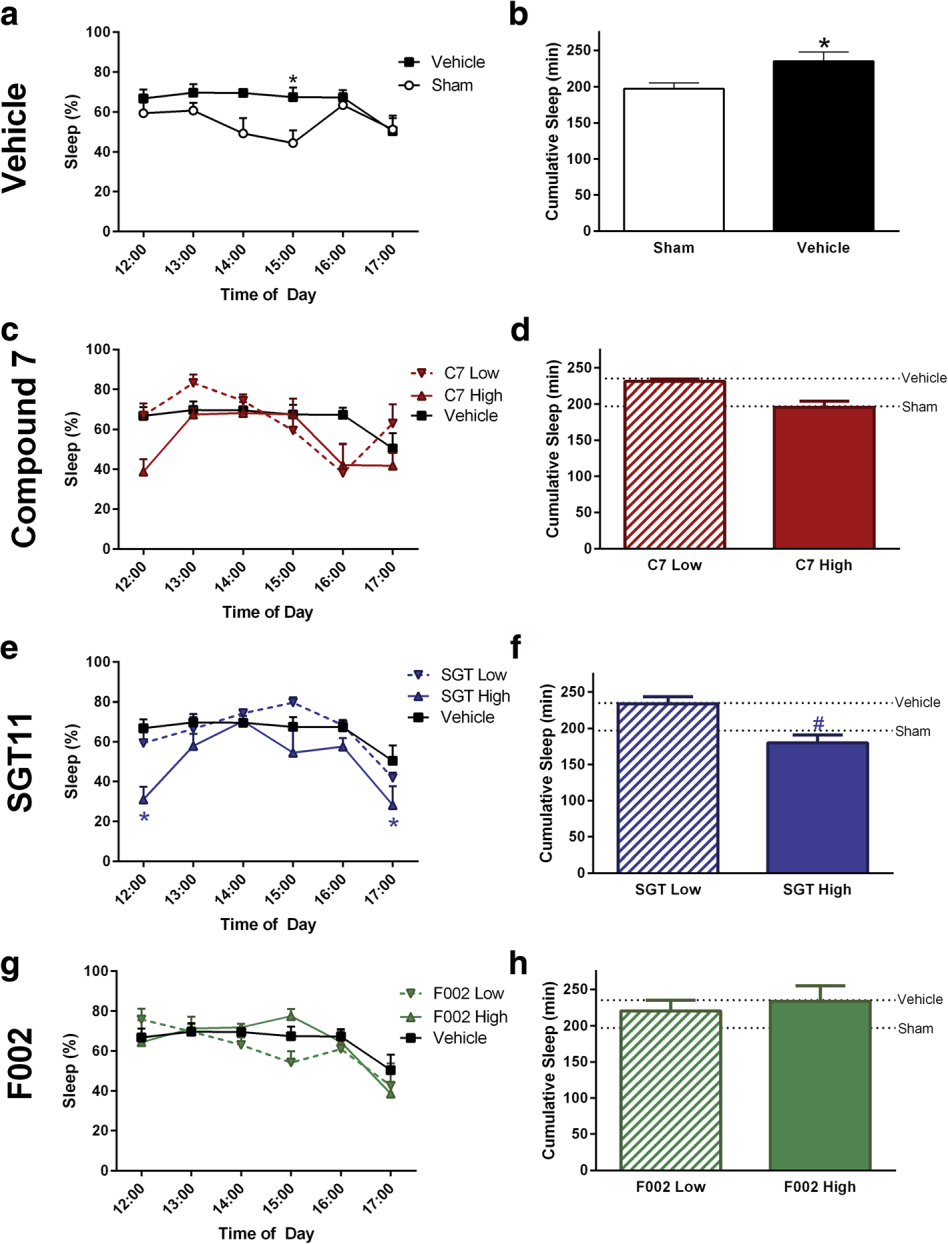
Screening of novel TNF-R1 inhibitors showed modulated post-traumatic sleep. Compounds were screened at two doses (high dose 20 mg/kg; low dose 2 mg/kg) for efficacy in modulating post-traumatic sleep compared to vehicle-treated and uninjured sham mice. Compounds were administered immediately following injury or sham procedure. A repeated measure two-way ANOVA was used to analyze main effect of treatment on percent sleep followed by Sidak’s multiple comparisons test when appropriate. Cumulative minutes slept was analyzed between sham and vehicle-treated using an un-paired *t* test, and across treatment groups using a one-way ANOVA followed by Tukey’s multiple comparisons test where appropriate. **a**, **b** Vehicle-treated brain-injured mice had a significant main effect of treatment on percent sleep (*F*_1,11_ = 6.835, *p* = 0.0241) and cumulative minutes spent sleeping (*t*_11_ = 2.614, *p* = 0.0241) compared to uninjured shams. **c**, **d** While both high- and low-dose compound C7 modulated post-traumatic sleep, there was no significant treatment effect between brain-injured mice treated with C7 compared to vehicle on percent sleep (*F*_2,9_ = 3.135, *p* = 0.0926) but there was an overall effect on cumulative minutes spent sleeping (*F*_3,15_ = 3.917, *p* = 0.0300). **e**, **f** There was an overall significant treatment effect between brain-injured mice treated with SGT compared to vehicle on percent sleep (*F*_2,10_ = 5.274, *p* = 0.0273) and cumulative minutes spent sleeping (*F*_3,16_ = 5.641, *p* = 0.0078). **g**, **h** Compound F002 did not significantly modulate percent sleep (*F*_2,10_ = 0.2786, *p* = 0.7625) or cumulative minutes spent sleeping (*F*_3,16_ = 2.252, *p* = 0.1217). Sleep groups; sham *n* = 7, vehicle-treated *n* = 6, C7-low *n* = 2, C7-high *n* = 4, SGT-low *n* = 4, SGT-high *n* = 3, F002-low *n* = 4, F002-low *n* = 3

In a preliminary cohort of mice, two doses of the three compounds were tested for their efficacy of modulating post-traumatic sleep, which is proposed to be a physiological bio-indicator of inflammation. As expected, diffuse TBI resulted in a significant increase in the percent of post-traumatic sleep over the first 6 h post-injury in vehicle-treated mice compared to uninjured shams [23] (Fig. 2a, b). While both doses of compound C7 modulated post-traumatic sleep compared to vehicle sleep profile, there were no significant differences compared to vehicle-treated brain-injured mice in the percent sleep during the first 6 h post-injury or the cumulative minutes slept (Fig. 2c, d). Both doses of compound SGT modulated the percent of post-traumatic sleep compared to vehicle-treated brain-injured mice (Fig. 2e). There was a significant decrease in the cumulative minutes slept by the SGT-treated brain-injured mice given a high dose, compared to the mice given a low dose (Fig. 2f). Compound F002 did not significantly modulate the percent of post-traumatic sleep, or cumulative minutes slept, regardless of the dose given (Fig. 2g, h). Uninjured sham mice showed no significant drug-induced change in sleep compared to baseline or the vehicle-treated group (Additional file 1: Figure S1). Using the modulation of post-traumatic sleep as an indicator of efficacy, compound C7 and SGT, but not F002, were selected for further study.

### Novel TNF-R1 inhibitors improved functional outcome measures following TBI

**Fig. 3:**
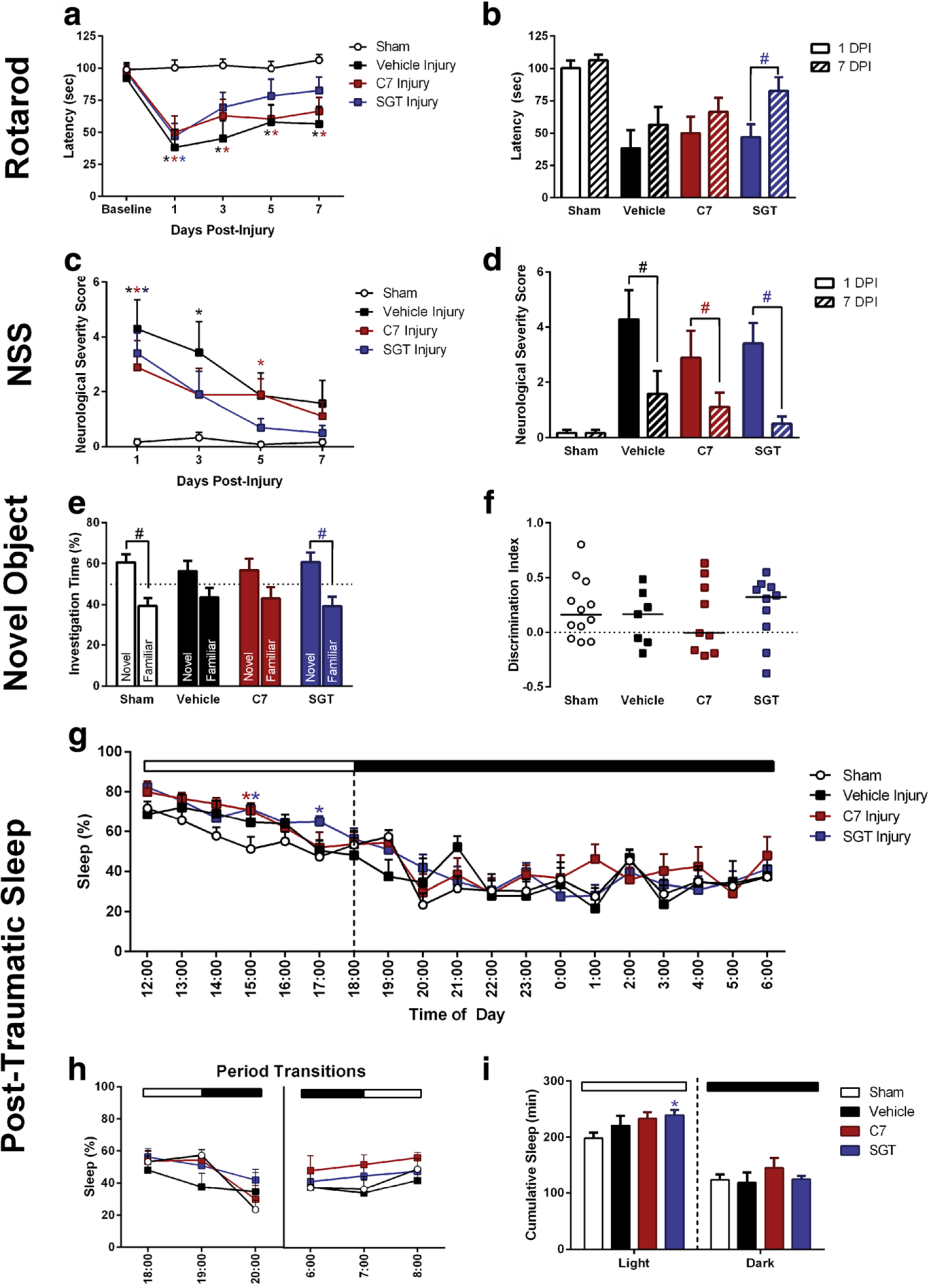
Novel TNF-R1 inhibitors modulated post-traumatic sleep and improved functional outcome measures following TBI. Following preliminary sleep screening, a second cohort of mice was tested for functional outcome following administration of low-dose (2 mg/kg) compounds given immediately following injury or sham procedure. **a** The rotarod was used to test motor function. There was a significant effect of time after injury (*F*_3,102_ = 9.612, *p* < 0.0001) and group effect on the latency to fall from the rotarod (*F*_3,34_ = 6.646, *p* = 0.0012). SGT attenuated motor deficits by 3 days post-injury (DPI). **b** There was a significant time effect (*F*_1,34_ = 17.14, *p* = 0.0002) and treatment effect (*F*_3,34_ = 8.450, *p* = 0.0002). Sidak’s post hoc analysis indicated SGT led to a significant improvement at 7 DPI compared to 1 DPI. **c** Neurological deficits were measured by a modified Neurological Severity Score (NSS). Median NSS scores differed among groups with a treatment effect (${\chi}_3^2$ = 32.669; *p* < 0.001) and time effect (${\chi}_3^2$ = 10.178; *p* = 0.021). At 3 and 5 DPI, SGT attenuated neurological deficits. **d** All injured groups showed improvement on the NSS task by 7 DPI. **e** Cognitive impairment was measured by novel object recognition (NOR). Differences in time spent exploring the novel versus familiar object revealed a significant difference between investigation times among sham (*t*_11_ = 2.686, *p* = 0.0212) and SGT-treated brain-injured mice (*t*_9_ = 2.277, *p* = 0.0488), indicating recall of the familiar object. Vehicle-treated brain-injured mice (*t*_6_ = 1.371, *p* = 0.2195) and C7 treated brain-injured mice (*t*_8_ = 1.259, *p* = 0.2435) spent similar times investigating both objects. **f** There were no significant differences in discrimination indices between groups (*F*_3,34_ = 0.2276, *p* = 0.8766). **g** TBI led to increased sleep during the first light cycle (*F*_3,25_) = 3.933, *p* = 0.0199). **h** TBI did not alter sleep during the transition from light to dark cycle (*F*_3,25_ = 0.5272, *p* = 0.6677). **i** There was an overall effect on cumulative sleep during the light cycle (*F*_3,25_) = 3.233, *p* = 0.0393) mice slept similarly during the dark cycle (*F*_3,25_ = 0.8089, *p* = 0.5009). Behavior groups: sham *n* = 12, vehicle-treated *n* = 7, C7-treated *n* = 9, SGT-treated *n* = 10. Sleep groups: sham *n* = 9, vehicle-treated *n* = 5, C7-treated *n* = 6, SGT-treated *n* = 9

To assess sensorimotor function, the rotarod task was used as previously published [22, 42, 46]. Motor function was tested as the latency to fall from the rotating rod out to 7 days post-injury, with significant effects for time post-injury and between treatment groups (Fig. 3a, b). By 3 days post-injury (DPI), SGT attenuated motor deficits compared to vehicle treatment (Fig. 3a) and increased latency to fall from the rod (Fig. 3b). Compound C7 showed no difference from the vehicle treatment group. Diffuse TBI led to immediate neurological deficits measured by a modified Neurological Severity Score (NSS) at 1, 3, and 5 DPI (Fig. 3c, d). At 3 and 5 DPI, SGT attenuated neurological deficits observed in vehicle-treated animals, and by 7 DPI, all injured groups recovered to uninjured sham performance, as expected. Cognitive function was measured using the novel object recognition task (Fig. 3e) [42]. SGT improved cognitive performance to uninjured sham levels, with SGT-treated brain-injured mice having a discrimination index higher than uninjured shams, indicating recall of the familiar object (Fig. 3f). To extend findings from the preliminary screening cohort, we measured sleep immediately following injury in the behavior cohort. TBI led to increased sleep during the first light cycle with C7-treated brain-injured mice sleeping significantly more at 15:00, and SGT-treated brain-injured mice sleeping significantly more at 15:00 and 17:00 (Fig. 3g). TBI did not significantly alter sleep during the transition from light to dark cycle (Fig. 3g, h). Following the light/dark change (19:00) sham mice, C7-treated, and SGT11-treated mice had an increase in sleep. Inversely, vehicle-treated mice had a decrease in sleep (Fig. 3h); however, this was not statistically significant. There were no alterations in sleep during the first dark cycle (Fig. 3h) and no changes during the transition from dark to light (Fig. 3h). Overall, SGT-treated brain-injured mice slept more cumulative minutes than uninjured shams during the light (sleep) cycle (Fig. 3i), but mice slept similarly during the dark (wake) cycle (Fig. 3i).

### Novel TNF-R1 inhibitors reduce neuroinflammation following TBI

**Fig. 4:**
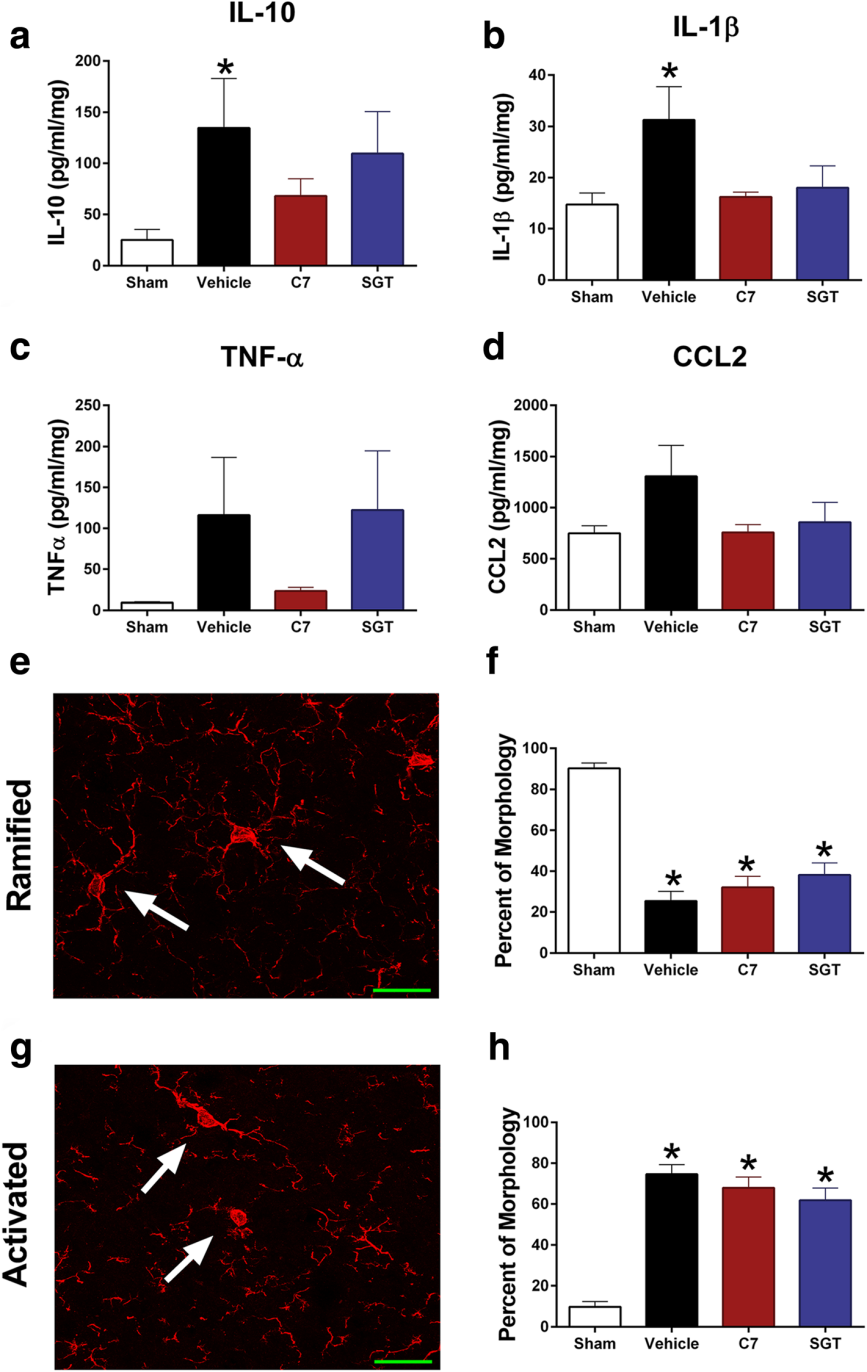
Novel TNF-R1 inhibitors reduce neuroinflammation following TBI. **a** Vehicle-treated mice, but not C7-treated or SGT-treated brain-injured mice, had significantly increased IL-10 (F_3,19_ = 3.217, *p* = 0.0460) and **b** IL-1β (F_3,19_ = 3.831, *p* = 0.0266) compared to uninjured shams at 3 h post-injury. **c** There was an injury-induced increase in TNF-α (*F*_3,19_ = 1.977, *p* = 0.1516) and **d** CCL2 (*F*_3,19_ = 2.340, *p* = 0.1057) but these increases failed to reach statistical significance. **e**, **f** Iba-1 immunohistochemistry revealed diffuse TBI significantly reduced ramified microglia in the cortex regardless of drug treatment (*F*_3,26_ = 33.57, *p* < 0.0001). Representative ramified microglia indicated by arrows. **g**, **h** All brain-injured groups had a significant increase in activated microglia compared to uninjured shams (*F*_3,26_ = 33.57, *p* < 0.0001). Representative activated microglia indicated by arrows. Cytokine groups: sham *n* = 9, vehicle-treated *n* = 5, C7-treated *n* = 4, SGT-treated *n* = 5. Immunohistochemistry groups: sham *n* = 7, vehicle-treated *n* = 7, C7-treated *n* = 8, SGT-treated *n* = 8; scale bar is 20 μm

Diffuse TBI increased inflammatory cytokines in the cortex at 3 h post-injury, and these increases were attenuated by treatment with C7 and SGT to levels that were not different from uninjured sham (Fig. 4a–d). Overall, diffuse TBI increased cortical levels of IL-10 and IL-1β in vehicle-treated brain-injured mice compared to uninjured shams (Fig. 4a, b), but not for cortical TNF-α and CCL2 levels (Fig. 4c, d). With the administration of either compound, cytokine levels were reduced to levels neither significantly elevated from uninjured sham nor lower than vehicle-treated brain-injured animals. Treatment with TNF-α receptor inhibitors did not prevent TBI-induced activation of microglia in the somatosensory cortex among brain-injured animals (Fig. 4e–h). All brain-injured groups demonstrated a reduced proportion of ramified microglia compared to uninjured sham mice at 7 DPI (Fig. 4e, f). All brain-injured groups demonstrated a greater proportion of activated microglia than uninjured sham mice (Fig. 4g, h).

## Discussion

Accumulated and published data indicate that inflammation contributes to secondary damage following TBI and therefore represents a plausible therapeutic target for intervention. Universally across rodent models, TNF-α levels (systemic and central) increase within hours of injury and return to basal levels as early as 24 h post-injury [24, 46]. TNF-α is an inflammatory cytokine that stimulates monocyte and glial activation and infiltration, neuronal and myelin loss, and blood-brain barrier permeability [47, 48]. TNFR-induced NF-ĸB activation can transcriptionally induce TNF and thus, amplify TNF and TNFR signaling pathways, including the expression of over 20 different cytokines and chemokines and receptors involved in immune regulation [49]. TNF-α binding to the TNF receptor can be blocked pharmacologically and thereby inhibit subsequent activation of downstream inflammatory signaling pathways. In the current study, three compounds (C7, SGT11, and F002) were synthesized to act as cavity-induced allosteric modifiers, binding to the TNF receptor and preventing downstream signaling. Since neuroinflammation and TNF signaling are pathophysiological hallmarks of clinical TBI [50], these studies set the stage for a pharmacological pipeline based on therapeutic biomarkers.

The use of sleep as a bio-indicator of inflammation is a novel approach to screen potential therapeutics. In the current study, we employ sleep as a valid indicator of inflammation. We have previously reported that acute sleep increases following experimental diffuse TBI and this increase is temporally associated with increased cytokine levels [17]. The current findings show diffuse TBI increased cumulative sleep, which was attenuated by C7 and SGT11. Furthermore, we were able to investigate two doses of each compound on their efficacy to modulate a physiological bio-indicator, sleep. We also showed diffuse TBI increased cortical cytokine levels, which was attenuated by C7 and SGT11. These data support the use of sleep as a bio-indicator of acute inflammation for screening potential therapeutic compounds, particularly anti-inflammatory compounds.

The novel TNFR1 inhibitors modulated inflammation via the TNFR1 signaling pathway, as predicted from molecular modeling. In vivo, compounds with efficacy to modulate post-traumatic sleep showed efficacy towards improved motor function following diffuse TBI. Our data are in line with pre-clinical studies using etanercept (Enbrel®), a biopharmaceutical marketed to treat autoimmune diseases by acting as a TNF inhibitor. Rats systemically injected with etanercept and subjected to fluid percussion brain injury demonstrated reduced motor and neurological deficits by 3 days post-injury, which was attributed to the attenuation of microglia activation and TNF production in the cortex, white matter, hippocampus, and hypothalamus [51]. A similar study using a weight-drop injury model indicated a single systemic injection of TNF-α synthesis inhibitor 3,6′-dithiothalidomide (DT) prevented injury-induced increases in TNF-α and ameliorated neuronal loss and cognitive impairments in the mouse [52]. DT injected in rats for 14 consecutive days has also been shown to reverse cognitive deficits induced by chronic inflammation [53]. A comprehensive review of studies using selective TNF inhibitors to treat brain injury and stroke has been previously published, for review see Tuttolomondo et al. [7].

TNF-α has pleiotropic actions in the brain. TNF-α can regulate host response to disease, control chemotaxis through the production of chemokines, regulate monocyte adhesion molecules, and contribute to microglia activation [7, 47]. As a potent pro-inflammatory cytokine, TNF promotes inflammatory signaling and injury-mediated microglial activation in the central nervous system [6]. In the current study, TNFR1 inhibiting compounds were used to prevent TNFR-induced NF-ĸB activation and the subsequent inflammatory surge [49], as demonstrated in vitro. We show a decrease in inflammatory cytokines in the cortex measured at 3 h post-injury, without a change in microglia activation between vehicle-treated brain-injured mice and brain-injured mice treated with C7 or SGT. We found C7 and SGT did not reduce microglia activation at 3 h post-injury in the cortex, possibly indicating that TNF inhibition was too transient to affect morphological inhibition of microglial activation, despite an impact on functional outcomes. Extended dosing strategies can test whether these novel compounds can modulate microglial activation. Although all measured cytokines and chemokines in the current study were reduced 3 h post-injury in brain-injured mice treated with C7 or SGT11 compared to vehicle, the decrease in TNF-α levels in the cortex did not reach significance. TNF-α stimulates the NF-ĸB signaling pathway directly, which can amplify inflammation and TNF production [49]. It is possible that TNF produced in response to TBI then induced NF-ĸB activation, leading to TNF transcription, thereby amplifying TNF and TNFR signaling pathways; these compounds would not necessarily inhibit TNF-induced TNF amplification, even though downstream inflammatory signals were dampened [11]. This positive auto-regulatory loop may overall affect TNF levels in the brain while other pro-inflammatory cytokines attenuate by the inhibition of TNFR-induced NF-ĸB activation.

Limitations of this study identified by the data require further support for (i) the role of sleep as predictive biomarker of underlying inflammation in the brain and (ii) the therapeutic efficacy of the candidate compounds. First, cumulative sleep was increased in SGT-treated brain-injured mice compared to uninjured shams across the 6-h light cycle immediately following injury, but at 3 h post-injury, we observed an attenuation of IL-1β, a sleep regulatory substance, in SGT-treated brain-injured mice. Also, the uninjured shams had a higher percent sleep than previously published from our lab [17], which may have reduced power to detect differences among groups. In the current study, the DMSO vehicle itself could have contributed to changes in sleep architecture [54]. Cavas et al. reported an increase in sleep following 15 and 20% DMSO (in saline) administration to rats [54]. Although the current study used 10% DMSO in saline as a solvent, this concentration may produce physiological alterations in sleep architecture in mice, and further investigation into the effects of DMSO on sleep in the mouse is necessary. We also note that TNF itself is a modulator of sleep. The combined sham group for this study included those treated with vehicle, C7, or SGT11, since sleep was not significantly different among groups. The experimental compounds could affect sleep architecture independent of injury-induced inflammation. To control for this, we analyzed the change in sleep after compound administration compared to baseline and the change from vehicle-treated sham cumulative minutes of sleep (Additional file 1: Figure S1). All three sham groups slept less in the post-surgery period compared to the baseline period, but this change was not dependent on the compound. Handling of the animals and surgical anesthesia likely contributed to the mice spending more time awake during the initial hours upon being returned to their cage. Although targeting TNF in the absence of TBI might influence sleep, uninjured sham mouse sleep was unaffected by the experimental compounds.

Further, the route and timing of administration requires further exploration to investigate clinical efficacy. We also acknowledge that the high dose contributed to the greatest modulation of post-traumatic sleep; however, availability and cost of the high dose warranted this exploratory study with low dose. Lastly, the increase in sleep could possibly be a result of decreased wakefulness. Orexin, a pleiotropic neuropeptide, is produced by neurons in the lateral hypothalamus and governs survival behaviors, including sleep/wake cycles, through the promotion of wakefulness [55]. A reduction in orexin-producing neurons could contribute to a reduction in wakefulness observed as an increase in sleep. Although we do not anticipate a loss in orexin neurons acutely at 6 h post-injury, further experiments are likely necessary to investigate brain injury-induced death of orexin neurons. To increase the efficiency of the proposed systematic pre-clinical approach, flow cytometry could report on microglial phenotypes in response to drug treatment as an alternate outcome measure. Using flow cytometry to quantify cells may prove advantageous over immunohistochemistry and cell count analyses.

## Conclusions

**Fig. 5:**
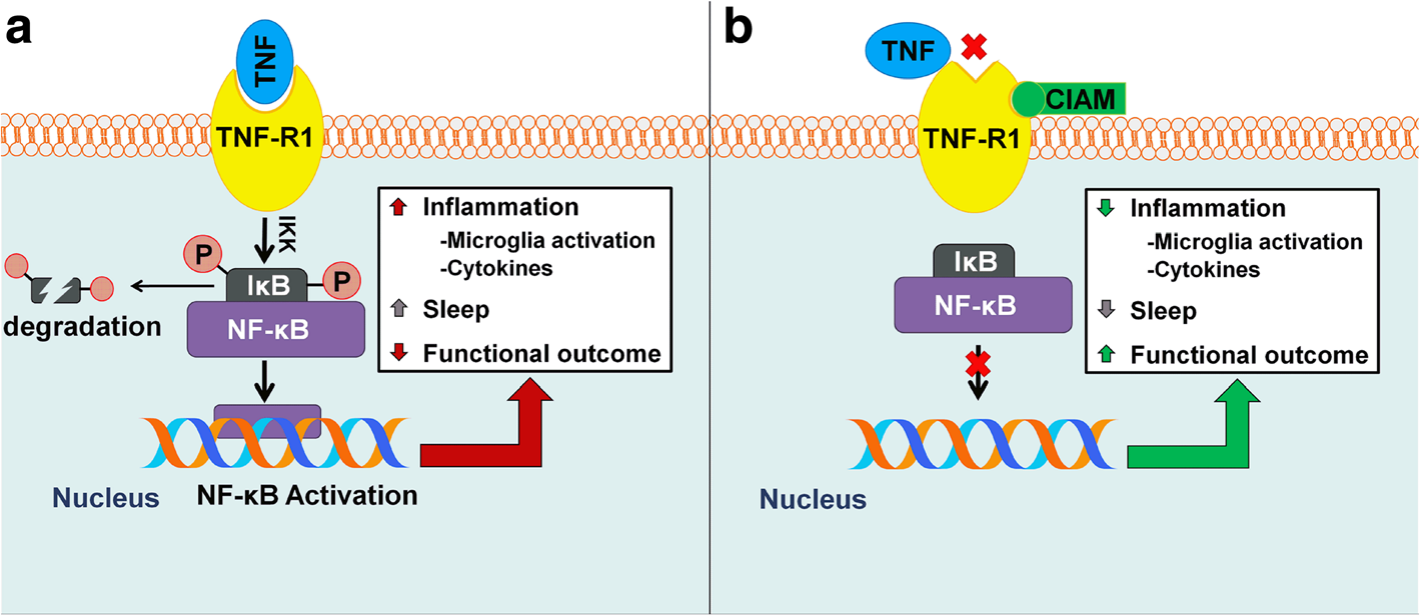
TNF-R1 inhibitors identified as potential therapeutic candidates for diffuse traumatic brain injury. **a** TNF binds the extracellular domain of TNF-R1 which leads to TNF-induced activation of NF-κB following the phosphorylation ubiquitination and degradation of inhibitor of κB (IκB) proteins, thus retaining NF-κB within the cytoplasm. NF-κB activation signals inflammatory pathways leading to an increase in cytokines and sleep, and a decrease in functional outcome. **b** Experimental cavity-induced allosteric modifiers (CIAMs) of TNF-R1 bind to the receptor and prevent TNF from binding. This prevents TNF-induced activation of NF-κB which dampens downstream inflammatory signaling reducing cytokine production, preventing inflammation-induced sleep, and improving functional outcome

These findings are consistent with improved functional outcome from brain injury by inhibiting TNFR-induced NF-ĸB signaling [7, 51–53, 56–59]. In concordance with previous reports, our results show an accelerated improvement in sensorimotor, neurological, and cognitive function when brain-injured mice are treated with compounds that inhibit TNFR1 (Fig. 5). On the basis of these observations, we add evidence that pharmacological inhibition of TNF-α is a plausible treatment for acute diffuse brain injury [56]. Furthermore, our results raise the consideration that post-traumatic sleep can be used as a bio-indicator of neuroinflammation and operate as a therapeutic biomarker; therefore, sleep could serve as a predictive biomarker of therapeutic efficacy. We posit that sleep should be considered as a valuable screening tool for the development and testing of drugs that target brain injury-induced inflammation in the laboratory and possibly the clinic.

## Additional file

Additional file 1: **Figure S1.** Uninjured sham mice showed no significant drug-induced change in sleep compared to baseline or the vehicle treated group. (A) There was no significant treatment effect on sleep in uninjured sham mice when compared to their pre-treatment baseline sleep (F_2,6_ = 0.6759, *p* = 0.5436). (B) The change in cumulative sleep from vehicle-treated shams was calculated and there was no significant treatment effect on sleep in the C7-treated or SGT-treated shams (t_4_ = 1.689, *p* = 0.1665). (TIF 152 kb)

## References

[1] Coronado VG, McGuire LC, Sarmiento K, Bell J, Lionbarger MR, Jones CD, Geller AI, Khoury N, Xu L (2012). Trends in traumatic brain injury in the U.S. and the public health response: 1995-2009. J Saf Res.

[2] Werner C, Engelhard K (2007). Pathophysiology of traumatic brain injury. Br J Anaesth.

[3] Janowitz T, Menon DK (2010). Exploring new routes for neuroprotective drug development in traumatic brain injury. Sci Transl Med.

[4] Ziebell JM, Morganti-Kossmann MC (2010). Involvement of pro- and anti-inflammatory cytokines and chemokines in the pathophysiology of traumatic brain injury. Neurotherapeutics.

[5] Morganti-Kossmann MC, Rancan M, Stahel PF, Kossmann T (2002). Inflammatory response in acute traumatic brain injury: a double-edged sword. Curr Opin Crit Care.

[6] McCoy MK, Tansey MG (2008). TNF signaling inhibition in the CNS: implications for normal brain function and neurodegenerative disease. J Neuroinflammation.

[7] Tuttolomondo A, Pecoraro R, Pinto A (2014). Studies of selective TNF inhibitors in the treatment of brain injury from stroke and trauma: a review of the evidence to date. Drug Des Devel Ther.

[8] Dong Y, Fischer R, Naude PJ, Maier O, Nyakas C, Duffey M, Van der Zee EA, Dekens D, Douwenga W, Herrmann A (2016). Essential protective role of tumor necrosis factor receptor 2 in neurodegeneration. Proc Natl Acad Sci U S A.

[9] Wang Y, Han G, Chen Y, Wang K, Liu G, Wang R, Xiao H, Li X, Hou C, Shen B (2013). Protective role of tumor necrosis factor (TNF) receptors in chronic intestinal inflammation: TNFR1 ablation boosts systemic inflammatory response. Lab Investig.

[10] Aggarwal BB (2003). Signalling pathways of the TNF superfamily: a double-edged sword. Nat Rev Immunol.

[11] Sedger LM, McDermott MF (2014). TNF and TNF-receptors: from mediators of cell death and inflammation to therapeutic giants—past, present and future. Cytokine Growth Factor Rev.

[12] Krueger JM (1993). Central cytokines and sleep. J Immunol.

[13] Krueger JM, Takahashi S, Kapas L, Bredow S, Roky R, Fang JD, Floyd R, Renegar KB, Guhathakurta N, Novitsky S, Obal F (1995). Cytokines in sleep regulation. Adv Neuroimmunol.

[14] Krueger JM, Obal F, Fang JD, Kubota T, Taishi P (2001). The role of cytokines in physiological sleep regulation. Ann N Y Acad Sci.

[15] Krueger JM (2008). The role of cytokines in sleep regulation. Curr Pharm Design.

[16] Krueger JM, Rector DM, Churchill L (2007). Sleep and cytokines. Sleep Med Clin.

[17] Rowe RK, Striz M, Bachstetter AD, Van Eldik LJ, Donohue KD, O'Hara BF, Lifshitz J (2014). Diffuse brain injury induces acute post-traumatic sleep. PLoS One.

[18] Kilkenny C, Browne WJ, Cuthill IC, Emerson M, Altman DG (2010). Improving bioscience research reporting: the ARRIVE guidelines for reporting animal research. PLoS Biol.

[19] Lifshitz J, Rowe RK, Griffiths DR, Evilsizor MN, Thomas TC, Adelson PD, McIntosh TK (2016). Clinical relevance of midline fluid percussion brain injury: acute deficits, chronic morbidities and the utility of biomarkers. Brain Inj.

[20] Rowe RK, Griffiths DR, Lifshitz J, Kobeissy HF, Dixon EC, Hayes LR, Mondello S (2016). Midline (central) fluid percussion model of traumatic brain injury. Injury models of the central nervous system: methods and protocols.

[21] Lifshitz J, Chen ZX J, Xu X-M, Zhang J (2008). Fluid percussion injury. Animal models of acute neurological injuries.

[22] Rowe RK, Harrison JL, O'Hara BF, Lifshitz J (2014). Recovery of neurological function despite immediate sleep disruption following diffuse brain injury in the mouse: clinical relevance to medically untreated concussion. Sleep.

[23] Rowe RK, Harrison JL, O'Hara BF, Lifshitz J (2014). Diffuse brain injury does not affect chronic sleep patterns in the mouse. Brain Inj.

[24] Harrison JL, Rowe RK, O'Hara BF, Adelson PD, Lifshitz J (2014). Acute over-the-counter pharmacological intervention does not adversely affect behavioral outcome following diffuse traumatic brain injury in the mouse. Exp Brain Res.

[25] Rowe RK, Griffiths DR, Lifshitz J (2016). Midline (central) fluid percussion model of traumatic brain injury. Methods Mol Biol.

[26] Hosseini AH, Lifshitz J (2009). Brain injury forces of moderate magnitude elicit the fencing response. Med Sci Sports Exerc.

[27] Rowe RK, Harrison JL, Thomas TC, Pauly JR, Adelson PD, Lifshitz J (2013). Using anesthetics and analgesics in experimental traumatic brain injury. Lab Anim (NY).

[28] Nakachi H, Aoki K, Tomomatsu N, Alles N, Nagano K, Yamashiro M, Zhang H, Murali R, Greene MI, Ohya K, Amagasa T (2012). A structural modulator of tumor necrosis factor type 1 receptor promotes bone formation under lipopolysaccharide-induced inflammation in a murine tooth extraction model. Eur J Pharmacol.

[29] Donohue KD, Medonza DC, Crane ER, O'Hara BF (2008). Assessment of a non-invasive high-throughput classifier for behaviours associated with sleep and wake in mice. Biomed Eng Online.

[30] Mang GM, Nicod J, Emmenegger Y, Donohue KD, O'Hara BF, Franken P (2014). Evaluation of a piezoelectric system as an alternative to electroencephalogram/ electromyogram recordings in mouse sleep studies. Sleep.

[31] McShane BB, Galante RJ, Jensen ST, Naidoo N, Pack AI, Wyner A (2010). Characterization of the bout durations of sleep and wakefulness. J Neurosci Methods.

[32] Ziebell JM, Bye N, Semple BD, Kossmann T, Morganti-Kossmann MC (2011). Attenuated neurological deficit, cell death and lesion volume in Fas-mutant mice is associated with altered neuroinflammation following traumatic brain injury. Brain Res.

[33] Semple BD, Bye N, Rancan M, Ziebell JM, Morganti-Kossmann MC (2010). Role of CCL2 (MCP-1) in traumatic brain injury (TBI): evidence from severe TBI patients and CCL2−/− mice. J Cereb Blood Flow Metab.

[34] Chen Y, Constantini S, Trembovler V, Weinstock M, Shohami E (1996). An experimental model of closed head injury in mice: pathophysiology, histopathology, and cognitive deficits. J Neurotrauma.

[35] Pleasant JM, Carlson SW, Mao H, Scheff SW, Yang KH, Saatman KE (2011). Rate of neurodegeneration in the mouse controlled cortical impact model is influenced by impactor tip shape: implications for mechanistic and therapeutic studies. J Neurotrauma.

[36] Han X, Tong J, Zhang J, Farahvar A, Wang E, Yang J, Samadani U, Smith DH, Huang JH (2011). Imipramine treatment improves cognitive outcome associated with enhanced hippocampal neurogenesis after traumatic brain injury in mice. J Neurotrauma.

[37] Ennaceur A, Aggleton JP (1997). The effects of neurotoxic lesions of the perirhinal cortex combined to fornix transection on object recognition memory in the rat. Behav Brain Res.

[38] Bachstetter AD, Xing B, de Almeida L, Dimayuga ER, Watterson DM, Van Eldik LJ (2011). Microglial p38alpha MAPK is a key regulator of proinflammatory cytokine up-regulation induced by toll-like receptor (TLR) ligands or beta-amyloid (Abeta). J Neuroinflammation.

[39] Cao T, Thomas TC, Ziebell JM, Pauly JR, Lifshitz J (2012). Morphological and genetic activation of microglia after diffuse traumatic brain injury in the rat. Neuroscience.

[40] Ziebell JM, Taylor SE, Cao T, Harrison JL, Lifshitz J (2012). Rod microglia: elongation, alignment, and coupling to form trains across the somatosensory cortex after experimental diffuse brain injury. J Neuroinflammation.

[41] Lifshitz J, Lisembee AM (2012). Neurodegeneration in the somatosensory cortex after experimental diffuse brain injury. Brain Struct Funct.

[42] Harrison JL, Rowe RK, Ellis TW, Yee NS, O'Hara BF, Adelson PD, Lifshitz J (2015). Resolvins AT-D1 and E1 differentially impact functional outcome, post-traumatic sleep, and microglial activation following diffuse brain injury in the mouse. Brain Behav Immun.

[43] R: a language and environment for stastical computing [http://www.cran.r-project.org//]. Accesed 19 Sept 2016.

[44] Signorell A (2016). DescTools: tools for descriptive statistics. 0.99.18 edition.

[45] Murali R, Cheng X, Berezov A, Du X, Schon A, Freire E, Xu X, Chen YH, Greene MI (2005). Disabling TNF receptor signaling by induced conformational perturbation of tryptophan-107. Proc Natl Acad Sci U S A.

[46] Bachstetter AD, Rowe RK, Kaneko M, Goulding D, Lifshitz J, Van Eldik LJ (2013). The p38alpha MAPK regulates microglial responsiveness to diffuse traumatic brain injury. J Neurosci.

[47] Chio CC, Lin MT, Chang CP (2015). Microglial activation as a compelling target for treating acute traumatic brain injury. Curr Med Chem.

[48] Rochfort KD, Cummins PM (2015). The blood-brain barrier endothelium: a target for pro-inflammatory cytokines. Biochem Soc Trans.

[49] Yamamoto Y, Gaynor RB (2001). Therapeutic potential of inhibition of the NF-kappaB pathway in the treatment of inflammation and cancer. J Clin Invest.

[50] Di Battista AP, Rhind SG, Hutchison MG, Hassan S, Shiu MY, Inaba K, Topolovec-Vranic J, Neto AC, Rizoli SB, Baker AJ (2016). Inflammatory cytokine and chemokine profiles are associated with patient outcome and the hyperadrenergic state following acute brain injury. J Neuroinflammation.

[51] Chio CC, Chang CH, Wang CC, Cheong CU, Chao CM, Cheng BC, Yang CZ, Chang CP (2013). Etanercept attenuates traumatic brain injury in rats by reducing early microglial expression of tumor necrosis factor-alpha. BMC Neurosci.

[52] Baratz R, Tweedie D, Wang JY, Rubovitch V, Luo W, Hoffer BJ, Greig NH, Pick CG (2015). Transiently lowering tumor necrosis factor-alpha synthesis ameliorates neuronal cell loss and cognitive impairments induced by minimal traumatic brain injury in mice. J Neuroinflammation.

[53] Belarbi K, Jopson T, Tweedie D, Arellano C, Luo W, Greig NH, Rosi S (2012). TNF-alpha protein synthesis inhibitor restores neuronal function and reverses cognitive deficits induced by chronic neuroinflammation. J Neuroinflammation.

[54] Cavas M, Beltran D, Navarro JF (2005). Behavioural effects of dimethyl sulfoxide (DMSO): changes in sleep architecture in rats. Toxicol Lett.

[55] Clark IA, Vissel B (2014). Inflammation-sleep interface in brain disease: TNF, insulin, orexin. J Neuroinflammation.

[56] Tobinick E, Kim NM, Reyzin G, Rodriguez-Romanacce H, DePuy V (2012). Selective TNF inhibition for chronic stroke and traumatic brain injury: an observational study involving 629 consecutive patients treated with perispinal etanercept. CNS Drugs.

[57] Baratz R, Tweedie D, Rubovitch V, Luo W, Yoon JS, Hoffer BJ, Greig NH, Pick CG (2011). Tumor necrosis factor-alpha synthesis inhibitor, 3,6′-dithiothalidomide, reverses behavioral impairments induced by minimal traumatic brain injury in mice. J Neurochem.

[58] Sun YX, Dai DK, Liu R, Wang T, Luo CL, Bao HJ, Yang R, Feng XY, Qin ZH, Chen XP, Tao LY (2013). Therapeutic effect of SN50, an inhibitor of nuclear factor-kappaB, in treatment of TBI in mice. Neurol Sci.

[59] Wang YX, You Q, Su WL, Li Q, Hu ZQ, Wang ZG, Sun YP, Zhu WX, Ruan CP (2013). A study on inhibition of inflammation via p75TNFR signaling pathway activation in mice with traumatic brain injury. J Surg Res.

